# Dyeing Industry Effluent System as Lipid Production Medium of *Neochloris* sp. for Biodiesel Feedstock Preparation

**DOI:** 10.1155/2014/529560

**Published:** 2014-08-27

**Authors:** Vidyadharani Gopalakrishnan, Dhandapani Ramamurthy

**Affiliations:** Fermentation Technology Lab, Department of Microbiology, Periyar University, Salem-11, Tamil Nadu 636011, India

## Abstract

Microalgae lipid feedstock preparation cost was an important factor in increasing biodiesel fuel hikes. This study was conducted with the concept of implementing an effluent wastewater as lipid production medium for microalgae cultivation. In our study textile dyeing industry effluent was taken as a lipid production medium for *Neochloris* sp. cultivation. The changes in physicochemical analysis of effluent before and after *Neochloris* sp. treatment were recorded using standard procedures and AAS analysis. There was especially a reduction in heavy metal like lead (Pb) concentration from 0.002 ppm to 0.001 ppm after *Neochloris* sp. treatment. *Neochloris* sp. cultivated in Bold Basal Medium (BBM) (specific algal medium) produced 41.93% total lipid and 36.69% lipid was produced in effluent based cultivation. Surprisingly *Neochloris* sp. cultivated in effluent was found with enhanced neutral lipid content, and it was confirmed by Nile red fluorescence assay. Further the particular enrichment in oleic acid content of the cells was confirmed with thin layer chromatography (TLC) with oleic acid pure (98%) control. The overall results suggested that textile dyeing industry effluent could serve as the best lipid productive medium for *Neochloris* sp. biodiesel feedstock preparation. This study was found to have a significant impact on reducing the biodiesel feedstock preparation cost with simultaneous lipid induction by heavy metal stress to microalgae.

## 1. Introduction

Algae comprise a wide variety of photosynthetic heterotrophic organisms that belong to different phylogenetic groups, denoting many taxonomic divisions. They are inhabited predominantly in fresh and marine water ecosystems. Among them microalgae are predominant in both marine and freshwater ecosystems. They contribute to more than half of the total primary production at the bottom level of the food chain [1]. Most of the microalgae can survive and proliferate in a wide range of environmental conditions due to their secondary metabolite acquisition. Thus microalgae gained more attention in the food, aquaculture, fuel, and biotechnology industries [2].

Environmental issue of industrial effluents and their concomitant deposition in discharging land and water system led to severe obstruction in the ecosystem. Particularly the heavy metal load in effluents is considered to be toxic and high level pollutants of water systems. The unknown exposure of these metals to humans leads to many hereditary diseases and cancer. In order to eliminate the adverse effects of these heavy metal constituents, proper treatment and recycling of the effluent are important. Also simultaneous reduction of other physicochemical parameters in effluent water that disturbs environmental stability should also be considered. Mostly the time consuming effluent treatment processes result in an economic crisis to the industrial sectors. Our natural system has its own candidates such as bacteria, fungi, and algae to remove these anthropogenic factors.

By conventional physical and chemical technologies such as membrane filtration, coagulation, precipitation, floatation, adsorption, ion exchange, chemical reduction, ultrasonic mineralization, electrolysis, and advanced chemical oxidation the effluents are treated. Some of these methods are termed to be effective; also they possess underlying drawbacks such as high cost, intensive energy requirements, and formation of hazardous byproducts and generation of sludge which causes secondary pollution [3]. In order to avoid these effects, specific attention should be given to biological treatment processes because of their cost-effective and ecofriendly characteristics. Next to bacteria, microalgae are important candidates in treating industrial effluents due to their ability to adapt in adverse conditions.

Generally algae lipids can be classified into two major groups, namely, the nonpolar lipids (acylglycerol, sterol, free fatty acids, hydrocarbons, wax, and steryl esters) and polar lipids (phosphoglycerides, glycosylglycerides) [4]. Each of the lipid constituents has its own important function. For instance, polar lipids and some sterols are playing an essential role in biological membrane construction. It helps in the membrane permeability and provides a barrier between organelles within cells. Also, they are involved in cell signaling pathways (e.g., inositol lipids, sphingolipids, and oxidative products of polyunsaturated fatty acids). The nonpolar lipids mainly triacylglycerols (TAG) serve as an abundant energy storing material which can be easily catalyzed to provide metabolic energy.

Enhancing microalgae lipid production is necessary to reduce the biodiesel feedstock cost and its sustainability. A number of green microalgae strains can accumulate large quantities of lipids under nutrient stress. They are appearing to be the sustainable source of biodiesel that has great potential to replace fossil diesel. The oil content of microalgae may range from 16 to 68% dry weight and the oil yield from microalgae can reach up to 136,900 L/ha compared to other plant crops, which range from 172 to 5950 L/ha [5]. Further optimization of culture conditions can enhance the lipid content of microalgae. Some of the microalgae members can accumulate triglycerides as storage lipid up to 70% dry weight under nitrogen starvation.

For instance* Neochloris oleoabundans* is a microalga that can accumulate lipid up to 54% in its total dry cell weight [5]. Under adverse conditions like nitrogen depletion microalgae cells are accumulating lipid due to their change in metabolic pathways. In such condition triacylglycerides (TAGs) are the more preferable lipid than any other types of lipid. Based on the favorable features* Neochloris *sp. is preferred for the present work. Instead of depleting nutrients we attempted to give heavy metal stress through industrial effluent based cultivation. Textile dyeing industry effluent was selected for the present work due to their heavy metal load. This study revealed that lead (Pb) content in effluent influenced TAGs storage in* Neochloris *sp. It was confirmed by thin layer chromatography (TLC) and Nile red fluorescence imaging and quantification. Further optimization of the strain with desirable parameters during effluent based cultivation will be helpful for increased TAG production.

## 2. Materials and Methods

### 2.1. Cultivation* Neochloris *sp

The* Neochloris *sp. AY2 strain was obtained from the Fermentation Technology Lab in the Department of Microbiology at the Periyar University, located in Salem district, Tamil Nadu, India. 5 mL culture was inoculated in 250 mL Bold Basal Medium (BBM) and incubated at 25°C under 1500 Lux white fluorescent light illumination for 21 days. The culture was examined in 400x magnification under a light microscope and its purity was confirmed [6].

### 2.2. Characterization of Effluent Sample

Textile dyeing industry effluent was collected from local yawn dyeing industry located in Karur district and the effluent sample physicochemical properties of the effluent sample were analyzed by the following APHA protocol published in 1994. Heavy metals were analyzed using atomic adsorption spectroscopy (AAS).

### 2.3. Screening of* Neochloris *sp. in Textile Dyeing Industry Effluent

From the grown* Neochloris *sp. 10 mL of culture was drawn initially, inoculated in 200 mL of sterile raw effluent, and kept at 25°C under 1500 Lux illumination for 21 days. The adaptation of* Neochloris *sp. was confirmed after 14 days by Evan's blue staining [7]. The effluent was observed for physicochemical change after cultivation with* Neochloris *sp.

### 2.4. Lipid Extraction and Characterization

#### 2.4.1. Total Lipid Extraction

The total lipid sample was extracted from algal culture by Bligh and Dyer method, 1957. 10 mL of algae suspension was centrifuged at 3,800 RPM for 10 minutes to obtain a concentrated algae paste. The recorded dry cell weight (DCW) of the biomass was determined gravimetrically after drying at 60°C [8]. The extracted lipids were quantified to know the lipid producing ability of the strain (Figure 4). The lower layer was filtered through a Whatman number 1 filter into a previously weighed glass vessel (*W*_1_) with the help of a glass syringe. The solvent was dried in a water bath at 98°C and the vessel was weighed again (*W*_2_). The lipid content of the sample was calculated using the following formula:

(1) $$\begin{array}{c} \text{Lipid}\;\text{content}=\frac{W_{1}-W_{2}}{W_{d}}\times 100\%. \end{array}$$

#### 2.4.2. Thin Layer Chromatography (TLC)

The crude lipid extract was analyzed through thin layer chromatography with the help of saturated solvents, namely, hexane : diethyl ether : acetic acid 60 : 40 : 1. After running the TLC plate neutral lipids were stained using iodine vapors and viewed in visible light [9].

#### 2.4.3. Nile Red Fluorescence Assay

The microalgal cells were stained with Nile red (Sigma Aldrich) as described by Chen et al., 2009 [6], for fluorescent spectroscopy analysis. The emissions were measured as a potential fluorescence unit (PFU) in every two-day interval. To the 3 mL of culture 15 *μ*L aliquot of 7.8 *μ*M Nile red dissolved in acetone was added. The emission wavelength was measured at 570 nm. Fluorescence intensity was recorded over a period of 30 seconds. The neutral lipid concentrations in live stained cells were estimated.

## 3. Results

### 3.1. *Neochloris *sp. Cultivation

**Figure 1:**
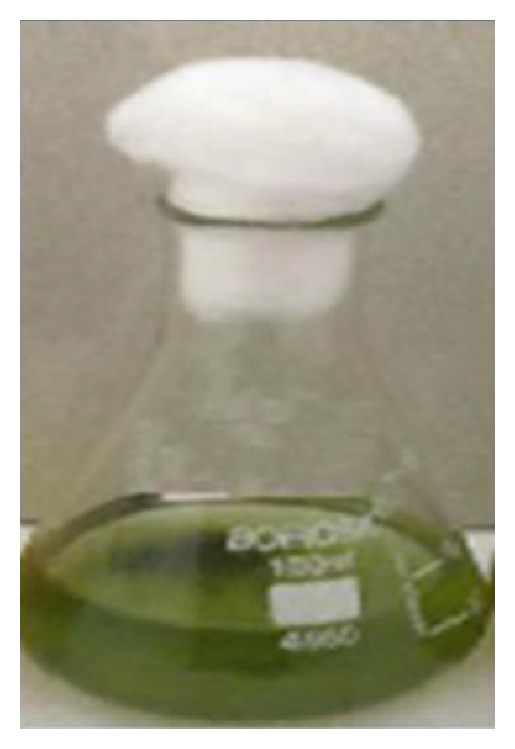
Grown culture of* Neochloris *sp. (after 21-day incubation).

**Figure 2:**
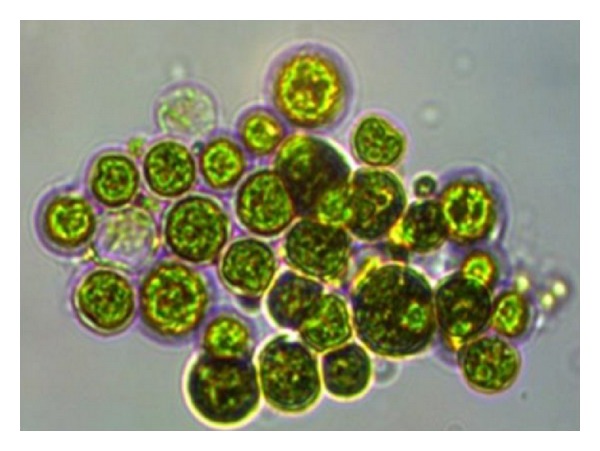
Microscopic appearance on* Neochloris *sp. in 1000x magnification under oil immersion.

The obtained* Neochloris *sp. was initially cultivated in 200 mL BBM (Figure 1) and kept at 25°C under 1500 Lux illumination for 21 days, and subsequent microscopic observations were performed to know the purity of the strain. The microscopic image (Figure 2) showed the microscopic appearance of* Neochloris *sp. cultivated in BBM after 21-day incubation. Biomass estimation studies were carried out initially and total lipids were extracted from the grown culture to know the native ability of the strain in lipid production.

### 3.2. Effluent Sample Characterizations

**Figure 3:**
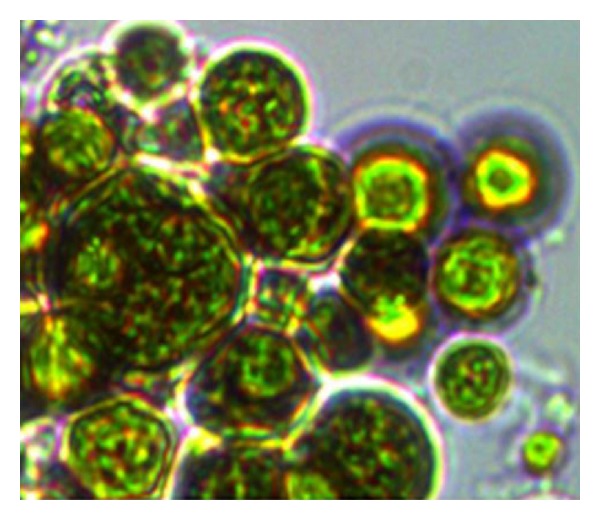
Microscopic appearance of Evan's blue stained* Neochloris *sp. under 400x illumination.

Textile effluent sample from local dyeing industry located in Karur district was stored in 4°C until processing. The physicochemical characterization was done for the effluent by standard APHA protocol [6]. Heavy metal analysis was performed with atomic absorption spectroscopy. The results were listed in Table 1. The strain was subjected to sterile 100% (raw) effluent; the adaptability was checked with the help of 1% Evan's blue dye. This is a good choice of stain for live-dead determination. Cells that contained intact semipermeable membrane (live) refused the dye and the dye penetrated the dead cells only. Therefore Evans's blue was referred to as a mortal strain rather than a vital strain [7] (Figure 3).

### 3.3. Total Lipid Extraction and Quantification

**Figure 4:**
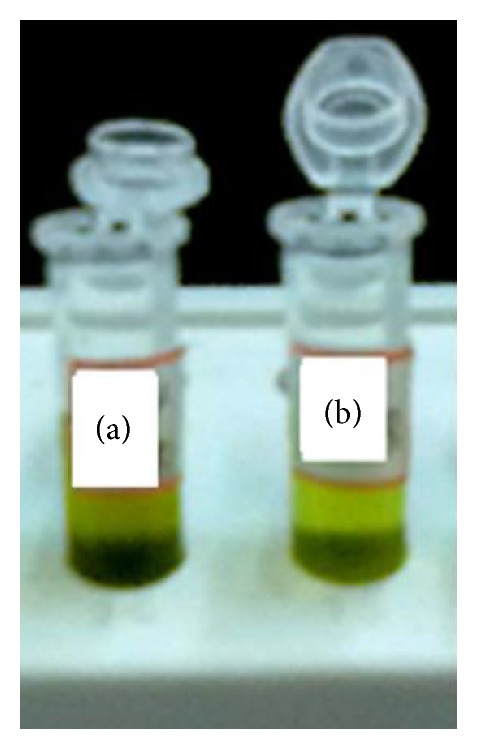
Total lipid extract: (a)* Neochloris *sp. cultivated in BBM and (b)* Neochloris *sp. cultivated in effluent.

Total lipid was extracted from* Neochloris *sp. (Figure 4) cultivated in BBM and* Neochloris *sp. cultivated in effluent.

According to the estimation, total lipid percentage was higher in BBM grown* Neochloris *sp. up to 41.93% than effluent grown* Neochloris *sp. that produced lipid up to 36.69% (Table 2). When considering total lipid productivity, BBM would be chosen as a suitable productive medium for lipid feedstock preparation.

### 3.4. Thin Layer Chromatography Analysis

The lipid extracts were analyzed using thin layer chromatography. The TLC plates were analysed (Figure 7) after spraying with iodine vapors for separated compounds. Iodine vapors were used to view nonpolar lipid such as triglyceride substances [9], and it was confirmed with appropriate standards. In total crude extract there was a peak, which showed the nearest Rf value for oleic acid 0.8, and it was confirmed by oleic acid standard peak.

### 3.5. Nile Red Fluorescent Spectroscopy Assay

**Figure 5:**
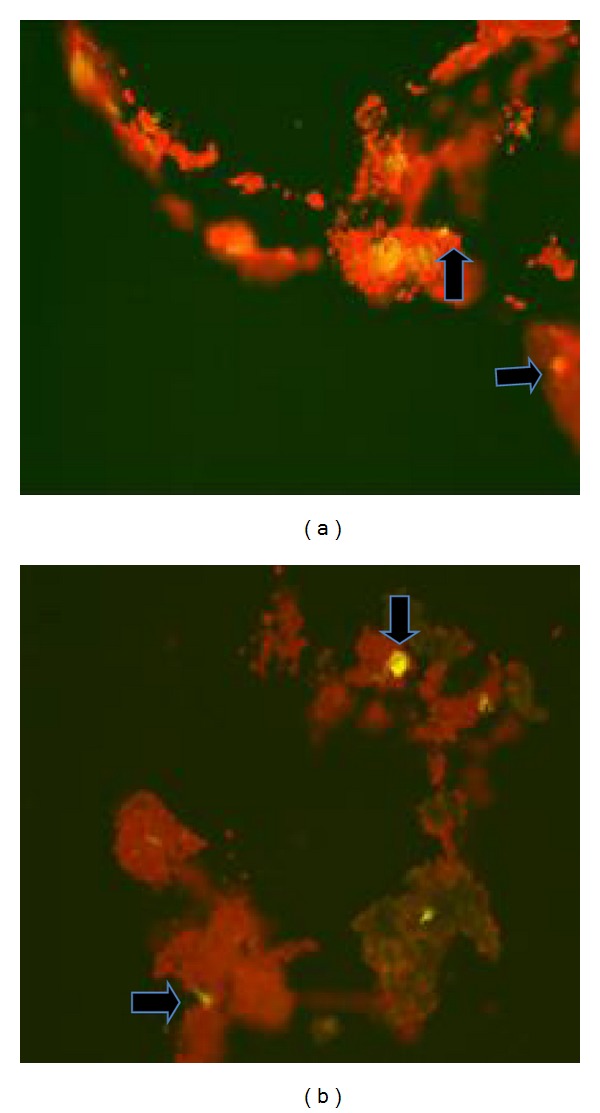
Nile red fluorescence microscopic images: (a)* Neochloris *sp. cells cultivated in effluent medium and (b)* Neochloris *sp. cells cultivated in BBM.

This method was quite helpful in confirming the presence of* in vivo* neutral lipid content and its estimation in microalgae cells (Figure 5). Based on the results the* Neochloris *sp. could be further optimized. The Nile red stained cells were observed for yellow fluorescent spots that denote neutral lipid under fluorescent microscopy [6]. The emission wavelength was measured at 570 nm, and the recorded potential fluorescent units from fluorescent spectroscopy were recorded for every two-day time interval.

**Figure 6:**
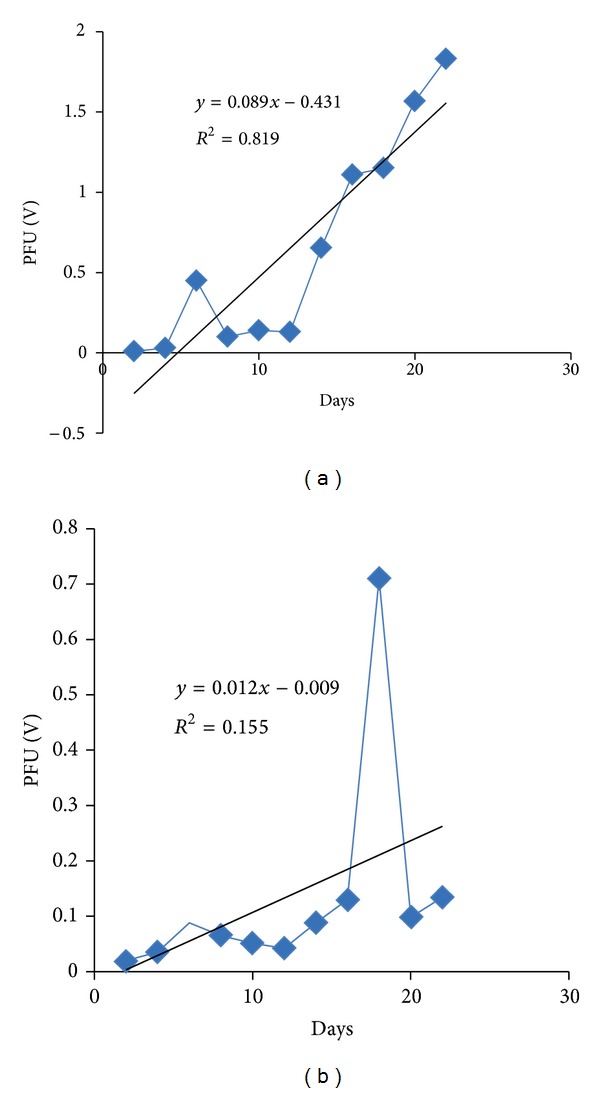
Nile red fluorescent assay based neutral lipid estimation. (a)* Neochloris *sp. cultivated in Effluent and (b)* Neochloris *sp. cultivated in BBM.

**Figure 7:**
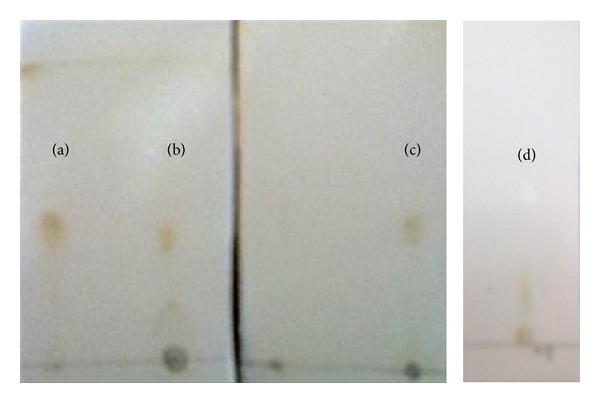
Thin layer chromatography plates for lipid extract from* Neochloris *sp. (a) Lipid from* Neochloris *sp. cultivated in effluent after 14 days, (b) lipid from* Neochloris *sp. cultivated in effluent after 21 days, (c) oleic acid control, and (d) lipid from* Neochloris *sp. cultivated in BBM after 21 days.

The fluorescence spectroscopy based Nile red assay was interpreted with the correlation value with the two variables such as PFU and the number of days. It gave an important notification on neutral lipid quantity inside the live* Neochloris *sp. In case of BBM grown* Neochloris *sp. the *R* ^2^ value is 0.155 (Figure 6(b)) and is highly negligible and denoted the less quantity of neutral lipid content. In effluent grown* Neochloris *sp. the *R* ^2^ value reached up to 0.819 (Figure 6(a)), and it denoted the increased neutral lipid content in the live algal cells.

## 4. Discussion

This study was started with the concept of implementing textile dyeing industry effluent as lipid production medium for microalgae. The adaptation of algae to different environmental conditions was reflected in an exceptional variety of lipids as well as a number of unusual compounds. Most of the algae could accumulate nonpolar lipid, especially in the form of TAG, a preferable biodiesel feedstock, up to 20–50% of dry cell weight [5]. This property gave an important position for algae in the renewable energy sector, and it was considered to be the potent source of biofuel. Lipids act as secondary metabolite in microalgae, maintaining specific membrane functions and cell signaling pathways while responding to the environment changes. Mostly TAG cellular accumulation corresponds to a shift in metabolism under unfavorable environmental conditions.

When compared to other industrial polluters textile dyeing industries are known to generate only a small fraction of total liquid effluent, but they may contribute a high proportion of total contaminants [10]. Textiles dyeing effluents was highly colored, contains saline, non biodegradable compounds, and with high oxygen demand (OD). It has been reported that the presence of metal and other dye compounds inhibits microbial activity and some cases that may cause failure of biological treatment systems.

Microalgae wastewater treatment was ecofriendly and offers the advantage of a cost-effective way of nutrient removal and biomass production. Textile dyeing industries are utilizing higher volume of water that finally results in an abundant wastewater generation; these are the major water polluting sources of ecosystem [11]. In 1974 EPA reported that the pollution parameters in textile wastewater effluents are suspended solids, BOD, COD, nitrogen, phosphate, temperature, and toxic chemicals such as phenolic compounds and heavy metals, pH, alkalinity, acidity, oils and grease, and sulfides. Still, it is an undoable task to treat textile effluent completely due to the presence of synthetic dyes that are difficult to remove by conventional methods.

These issues were taken into account and our study was targeted to treat the textile dyeing industry effluent for* Neochloris *sp. cultivation. This study was reported to be the first study on screening of* Neochloris *sp. in textile dyeing industry effluent for lipid production.

The following changes were recorded before and after* Neochloris *sp. cultivation in textile dyeing industry effluent. The pH was recorded as 10.3 initially which was an unsupportive factor for microalgae growth, and still we made an attempt to cultivate microalgae. Later on the pH was reduced up to 7.9 after 21 days of* Neochloris *sp. cultivation. Physicochemical properties were compared in between treated and untreated effluents and mentioned as follows. Likewise the total dissolved solids which were at 3240 ppm in raw effluent were reduced to 994 ppm, and total suspended solids which were at 804 ppm were reduced to 236 ppm. Initial total hardness was 1992 ppm that reduced to 581 ppm. Total alkalinity was 1025 ppm and reduced to 193 ppm. Dissolved oxygen was in the range of 13.2 ppm that was raised up to 19.7 ppm. Biological oxygen demand (BOD) at 923 ppm was reduced to 225 ppm. Chemical oxygen demand (COD) at 5680 ppm was tremendously reduced to 1962 ppm. The AAS analysis report for untreated effluent revealed the presence of lead (Pb) at 0.002 ppm, chromium (Cr) 0.005 ppm, and nickel (Ni) 0.001. Other heavy metals such as zinc, cadmium, copper, and aluminium were absent in the effluent sample. In treated effluent, heavy metal lead (Pb) was reduced to 0.001 ppm, whereas other heavy metal reduction was not observed.

Also the tolerance level of the* Neochloris *sp. for lead (Pb) concentration of 0.002 ppm was verified using Evan's blue staining. This staining method revealed the viability of* Neochloris* sp. in textile dyeing industry effluent. The hazardous parameters, color, and pH were reduced after* Neochloris *sp. treatment of the effluent. In our study we have concentrated on neutral lipid induction, and especially we found to know the reduction in Pb level from 0.002 to 0.001 ppm and increased neutral lipid content of* Neochloris *sp., the mechanism was still unknown. Further Nile red assay confirmed the neutral lipid analysis. The total lipid produced when* Neochloris *sp. was grown on BBM was 41.93% and the effluent grown* Neochloris *sp. produced lipid up to 36.69%.

The Nile red fluorescence assay was quite helpful in neutral lipid estimation. The potential fluorescence unit (PFU) measurement was correlated with the number of incubation days in cultivation medium. The calculated *R* ^2^ value for* Neochloris* sp. cultivated in BBM was 0.155 that directly stated the less significance with the incubation period and the neutral lipid content. It denoted the lower concentration of neutral lipid content inside the cell when cultivated in BBM. Oppositely the *R* ^2^ value for* Neochloris* sp. cultivated in the effluent was 0.819 and stated the high relevance of the effluent medium and incubation period provided in the respective change in neutral lipid content. It indirectly denoted that some of the components in the effluent system lead to an increase in neutral lipid content.

The heavy metals such as cadmium, iron, copper, and zinc were reported previously to increase the lipid content in many microalgae. For instance, cadmium treated cells of* Euglena gracilis* under illumination were found with increased lipid production [12]; likewise the effect of iron on* Chlorella vulgaris* was studied by Liu et al., 2008 [13], who reported that total lipid content was raised up to 56.6% in total biomass. The exact mechanism was still unclear.

Further studies on lipid characterization with thin layer chromatogram revealed that the effluent grown strain can accumulate oleic acid rather than BBM grown strain. It was confirmed with the oleic acid (98% pure) control. After obtaining this result, it had been reviewed with the physicochemical properties of the processed effluent. The changes before and after treatment with* Neochloris *sp. were measured; among four heavy metals that were presented in effluent medium only lead (Pb) was utilized by the strain. The initial concentration of 0.002 in ppm was reduced to 0.001 in ppm. This was considered to be the upliftment in the neutral lipid content of the effluent grown cells, especially the oil and acid content of the cells. It has been previously reported that oleic acid content of the algal cells can be influenced by lead (Pb). This result was added as a supportive point for utilizing textile dyeing industry effluent as lipid production medium for* Neochloris *sp. Only drawback was the reduction in total biomass after exponential phase. In addition, concern should be given to maintain the same growth rate of* Neochloris *sp. towards producing high amounts of neutral lipid in effluent based medium. Also the complementary advantage of the industrial sector was the recycled water from the effluent that could be reused in the same industry. In overview this study reported that textile dyeing industry effluent could be the best lipid productive medium for* Neochloris *sp. biodiesel feedstock preparation.

## Figures and Tables

**Table 1:** Physicochemical parameters of the textile dyeing industry effluent before and after treatmentwith *Neochloris *sp.

S. number	Parameter and heavy metals	Experimental value textile dyeing industry effluent	
		Untreated with *Neochloris *sp.	Treated with *Neochloris* sp.
1.	pH	10.3	7.9
2.	Total dissolved solids	3240 (ppm)	990 (ppm)
3.	Total suspended solids	804 (ppm)	236 (ppm)
5.	Total hardness	1992 (ppm)	581 (ppm)
6.	Total alkalinity	1025 (ppm)	193 (ppm)
8.	Dissolved oxygen	13.2 (ppm)	19.7 (ppm)
9.	BOD	923 (ppm)	225 (ppm)
10.	COD	5680 (ppm)	1962 (ppm)
11.	Pb	0.002 (ppm)	0.001 (ppm)
12.	Cr	0.005 (ppm)	0.005 (ppm)
13.	Ni	0.001 (ppm)	0.001 (ppm)
14.	Zn	0.006 (ppm)	0.006 (ppm)
15.	Cd	0.000 (ppm)	0.000 (ppm)
16.	Cu	0.000 (ppm)	0.000 (ppm)
17.	Al	0.000 (ppm)	0.000 (ppm)
18.	Hg	0.000 (ppm)	0.000 (ppm)

**Table 2:** Table showing the total lipid percentage in *Neochloris *sp.

S. number	Microalgae strain	Cultivation medium	Dry cell weight (DCW) (in g/L)	Lipid filtrate weight (*W*_1_) (g/L)	Dry lipid weight (*W*_2_) (g/L)	Total lipid content (in %)
1.	*Neochloris *sp.	BBM	88	0.93	0.561	41.93
2.	*Neochloris *sp.	Effluent sample I	109	2.7	2.3	36.69

## References

[1] Guschina IA, Harwood JL (2006). Lead and copper effects on lipid metabolism in cultured lichen photobionts with different phosphorus status. *Phytochemistry*.

[2] Richmond A (2004). *Handbook of Microalgal Culture: Biotechnology and Applied Phycology*.

[3] Robinson T, McMullan G, Marchant R, Nigam P (2001). Remediation of dyes in textile effluent: a critical review on current treatment technologies with a proposed alternative. *Bioresource Technology*.

[4] Gunstone FD, Harwood JL, Dijkstra AJ (2007). *The Lipid Handbook*.

[5] Chisti Y (2007). Biodiesel from microalgae. *Biotechnology Advances*.

[6] Chen W, Zhang C, Song L, Sommerfeld M, Hu Q (2009). A high throughput Nile red method for quantitative measurement of neutral lipids in microalgae. *Journal of Microbiological Methods*.

[7] Crippen RW, Perrier JL (1974). The use of neutral red and Evans blue for live-dead determinations of marine plankton. *Stain Technology*.

[8] Dalrymple OK, Halfhide T, Udom I (2013). Wastewater use in algae production for generation of renewable resources: a review and preliminary results. *Aquatic Biosystems*.

[9] Shah GC, Patidar A, Urkude V Analysis and Characterization of Algal Oil by Using Different Chromatographic Techniques for the Higher Production of Biodiesel from Scenedesmus Dimorphus Algal Species.

[10] Wang L, Li Y, Chen P (2010). Anaerobic digested dairy manure as a nutrient supplement for cultivation of oil-rich green microalgae *Chlorella* sp.. *Bioresource Technology*.

[11] Nemerow NL (1978). *Industrial Water Pollution: Origins, Characteristics and Treatment*.

[12] Einicker-Lamas M, Mezian GA, Fernandes TB (2002). *Euglena gracilis* as a model for the study of Cu^2+^ and Zn^2+^ toxicity and accumulation in eukaryotic cells. *Environmental Pollution*.

[13] Liu ZY, Wang GC, Zhou BC (2008). Effect of iron on growth and lipid accumulation in *Chlorella vulgaris*. *Bioresource Technology*.

